# The Effect of MoS_2_ and Si_3_N_4_ in Surface Plasmon Resonance Biosensors for HIV DNA Hybridization Detection: A Numerical Study

**DOI:** 10.3390/mi16030295

**Published:** 2025-02-28

**Authors:** Talia Tene, Diana Coello-Fiallos, María de Lourdes Palacios Robalino, Fabián Londo, Cristian Vacacela Gomez

**Affiliations:** 1 Department of Chemistry, Universidad Técnica Particular de Loja, Loja 110160, Ecuador; 2 Facultad de Ciencias, Escuela Superior Politécnica de Chimborazo (ESPOCH), Riobamba 060155, Ecuador; 3 INFN-Laboratori Nazionali di Frascati, Via E. Fermi 54, 00044 Frascati, Italy

**Keywords:** surface plasmon resonance, HIV DNA hybridization, Kretschmann configuration, transfer matrix method, silicon nitride, molybdenum disulfide

## Abstract

This study presents a numerical investigation of surface plasmon resonance (SPR) biosensors incorporating silicon nitride (Si_3_N_4_) and molybdenum disulfide (MoS_2_) for HIV DNA hybridization detection. By optimizing the thickness of Ag and Si_3_N_4_ and the number of MoS_2_ layers, two configurations, Sys_2_ (Ag-Si_3_N_4_) and Sys_3_ (Ag-Si_3_N_4_-MoS_2_), were selected for comparative analysis. Performance metrics, including the resonance angle shift, sensitivity, detection accuracy, and quality factor, demonstrated that Sys_2_ achieved the highest sensitivity of 210.9°/RIU and an enhanced figure of merit (86.98 RIU^−1^), surpassing state-of-the-art SPR sensors. Although Sys_3_ exhibited a lower sensitivity of 158.1°/RIU due to MoS_2_-induced optical losses, it provided a lower limit of detection, suggesting a trade-off between sensitivity and spectral broadening. Compared to previous SPR biosensors, the proposed configurations achieve superior sensitivity while maintaining stability and selectivity, positioning them as promising candidates for next-generation nucleic acid detection platforms.

## 1. Introduction

Detecting HIV DNA hybridization is fundamental for diagnosing and monitoring human immunodeficiency virus (HIV) infections, particularly in early detection and antiretroviral therapy (ART) management [1]. As a retrovirus, HIV primarily targets the immune system [2], and its detection through DNA hybridization refers to the identification of complementary single-stranded DNA (ssDNA) sequences that indicate the virus’s presence in biological samples [3]. During infection, viral RNA undergoes reverse transcription, producing complementary proviral DNA that integrates into the host genome, serving as a crucial genetic marker of both latent and active infections [4].

Conventional nucleic acid detection techniques such as polymerase chain reaction (PCR) [5], loop-mediated isothermal amplification (LAMP) [6], and enzyme-linked immunosorbent assay (ELISA) [7] provide high specificity for detecting HIV DNA hybridization. However, these methods typically require specialized laboratory infrastructure, costly reagents, and trained personnel, limiting their feasibility for point-of-care (POC) diagnostics.

Among the various biosensing strategies, optical detection techniques have gained prominence due to their ability to deliver direct, rapid, and quantitative results without requiring extensive sample preparation [8,9]. Surface plasmon resonance (SPR) biosensors have emerged as a particularly promising approach owing to their exceptional sensitivity to molecular interactions and their ability to function without the need for fluorescent or electrochemical labels [10,11]. Unlike electrochemical biosensors, which depend on electron transfer reactions, SPR exploits the interaction between incident light and collective electron oscillations at a metal-dielectric interface [12].

To further emphasize this, fluorescence and electrochemical biosensors have been widely used for HIV DNA hybridization detection [10,11,12], but both present limitations that SPR overcomes. For instance, fluorescence-based sensors require labeling, are prone to photobleaching, and involve complex optical setups, making them less practical for real-time, point-of-care applications. Electrochemical biosensors, while label-free, rely on redox reactions and electrode modifications, which can introduce chemical instability and signal variability. In contrast, SPR biosensors enable real-time, label-free detection by directly measuring refractive index changes upon DNA hybridization, eliminating the need for additional reagents.

A widely used configuration for SPR-based detection is the Kretschmann setup, which employs total internal reflection (TIR) within a high-refractive-index prism to efficiently excite surface plasmons [13,14]. At a specific incident angle, known as the resonance angle, the energy from the incident light is transferred to the surface plasmons, producing a characteristic dip in reflected light intensity. This angle is highly sensitive to changes in the refractive index at the sensor surface, making it particularly effective for detecting biomolecular interactions, including HIV DNA hybridization [15]. As complement to ssDNA strands hybridizing to form double-stranded DNA (dsDNA), the local refractive index increases, shifting the resonance angle, which serves as a quantifiable detection signal [8].

Traditional SPR sensors commonly use gold (Au) [16] and silver (Ag) [17] thin films as plasmonic materials due to their ability to sustain strong plasmonic excitations in the visible and near-infrared spectral ranges. However, these noble metals suffer from several inherent limitations, including broad resonance linewidths, high Ohmic losses, and chemical instability [18]. To mitigate these challenges, researchers have turned to alternative plasmonic and dielectric materials to enhance the sensitivity, stability, and spectral resolution of SPR-based biosensors.

Hence, this study proposes an advanced SPR biosensor that incorporates silicon nitride (Si_3_N_4_) and molybdenum disulfide (MoS_2_) to improve the detection of HIV DNA hybridization. Si_3_N_4_ is a high-refractive-index dielectric material that exhibits low optical loss, excellent chemical stability, and broad spectral transparency, making it highly suitable for optical biosensing applications [19]. Furthermore, Si_3_N_4_ is fully compatible with complementary metal-oxide-semiconductor (CMOS) technology, the dominant fabrication platform for modern microelectronics and photonics, allowing for the potential integration of SPR sensors into miniaturized, cost-effective photonic circuits [20,21]. This compatibility offers a pathway toward scalable manufacturing for lab-on-a-chip biosensing applications [22].

In addition to Si_3_N_4_, MoS_2_, a two-dimensional (2D) transition metal dichalcogenide (TMD), has gained attention for its unique optical and electronic properties that can further enhance SPR sensor performance [23,24]. Unlike conventional plasmonic metals, MoS_2_ exhibits a layer-dependent bandgap, which ranges from approximately 0.3 eV (bulk) to 2.0 eV (monolayer), making it highly adaptable for optimizing sensor response across various wavelengths [25]. Additionally, MoS_2_ demonstrates strong anisotropic optical behavior, meaning that its response varies based on crystal orientation, enabling highly confined surface plasmon polaritons (SPPs) with lower propagation losses, ultimately improving detection sensitivity [26].

Several key parameters are considered to evaluate the sensor’s performance in detecting HIV DNA hybridization, including sensitivity, the full width at half maximum (FWHM), the quality factor (QF), the figure of merit (FoM), the limit of detection (LoD), and the detection accuracy (DA). To systematically assess the performance of the proposed Si_3_N_4_–MoS_2_-based SPR sensor, this study employs the Transfer Matrix Method (TMM) as a computational framework for modeling multilayer optical systems [27]. Our findings provide a robust theoretical foundation for future experimental validation and demonstrate the potential for practical applications in HIV diagnostics, biosensing, and nucleic-acid-based disease detection.

## 2. Materials and Methods

### 2.1. Theoretical Framework

**Scheme 1:**
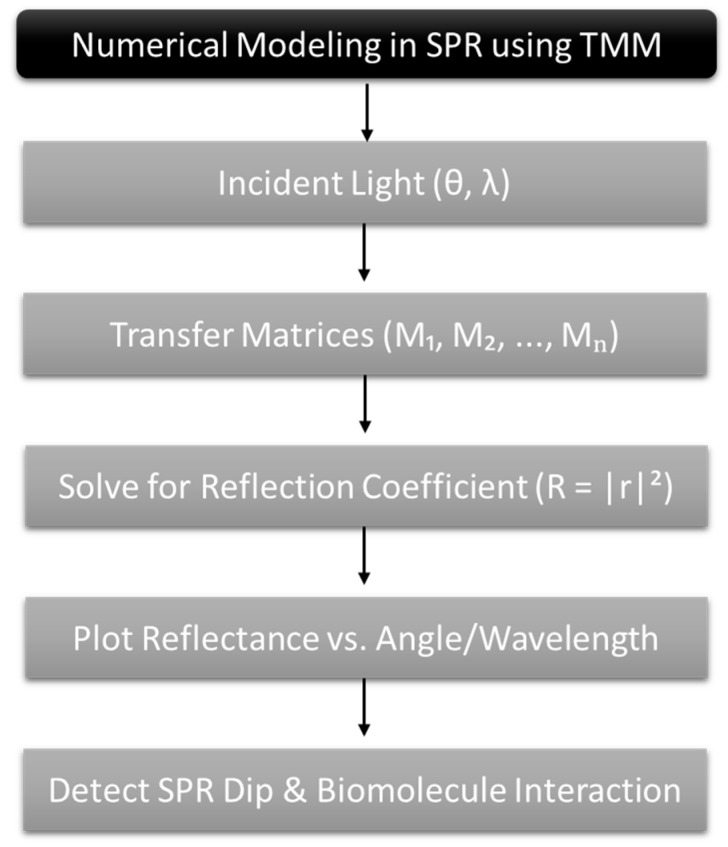
Illustration of numerical modeling approach using the TMM in SPR sensors.

The reflective intensity of the proposed *Nth*-layer sensor model is calculated using the TMM approach [28,29,30] (Scheme 1). The analysis of the sensor considers boundary conditions for the tangential component, with the initial limit of Z = Z_1_ = 0, and the final limit of Z_n−1_, giving the following expression:

(1) $$\left[\begin{array}{c} E_{1}\\ H_{1} \end{array}\right]=M_{ij}\left[\begin{array}{c} E_{N-1}\\ H_{N-1} \end{array}\right]$$

In Equation (1), *E*_1_, *E_N_*_−1_, *V*_1_, and *V_N_*_−1_ represent the tangential components of the electric and magnetic fields for the initial and *Nth* layers, respectively. *M_ij_* indicates the transfer matrix characteristics of the *Nth*-layer model, computed as:

(2) $$M_{ij}={\left[\prod_{k=2}^{N-1}M_{k}\right]}_{ij}=\left[\begin{array}{cc} M_{11} & M_{12}\\ M_{21} & M_{22} \end{array}\right]$$

With

(3) Mk=cos⁡βk(−i sin⁡βk)/qk−i qk sin⁡βkcos⁡βk

Denoting

(4) $$\beta_{k}=\frac{2\pi d_{k}}{\lambda_{0}}\sqrt{\varepsilon_{k}-n_{1}^{2}\sin^{2}\theta }$$

And

(5) $$q_{k}=\frac{\sqrt{\varepsilon_{k}-n_{1}^{2}\sin^{2}\theta }}{\varepsilon_{k}}$$

In Equations (3)–(5):

- $\lambda_{0}$ represents the wavelength of the incident light,
- $n_{1}$ is the refractive index,
- $\varepsilon_{k}$ represents the dielectric constant,
- $\beta_{k}$ represents the phase constant,
- θ represents the entrance angle,
- $d_{k}$ represents the depth of the $k^{th}$ layer.

For comparison with experiments, we adopt the use of an He-Ne laser with $\lambda_{0}=633$ nm. After straightforward computations, the total reflection of the *Nth*-layer model can be expressed as:

(6) $$R={\left|\frac{\left(M_{11}+M_{12}\;q_{N}\right)q_{1}-\left(M_{21}+M_{22}\;q_{N}\right)}{\left(M_{11}+M_{12}\;q_{N}\right)q_{1}+\left(M_{21}+M_{22}\;q_{N}\right)}\right|}^{2}$$

By using Equation (6), the reflectance as a function of the angle of incidence (SPR curve) can be calculated.

### 2.2. Performance Metrics

We now move on to the main performance metric of the proposed sensors [19,31,32]. The first parameter is the sensitivity enhancement regarding the baseline sensors after/before pathogen adsorption, denoted as:

(7) $$∆S_{RI}^{after}=\frac{\left(S_{RI}^{after}-S_{RI}^{before}\right)}{S_{RI}^{before}}$$

Then, the sensitivity to the refractive index change can be expressed as:

(8) $$S_{RI}=\frac{∆\theta }{∆n}$$

Here, ∆θ represents the angle shift variation and ∆n represents the refractive index variation. The detection accuracy (DA) can be expressed as in terms of ∆θ and the full width at half maximum (FWHM) of the SPR curve, as:

(9) $$DA=\frac{∆\theta }{FWHM}$$

The quality factor (QF) can be expressed in terms of $S_{RI}$ and FWHM, as follows:

(10) $$QF=\frac{S_{RI}}{FWHM}$$

The figure of merit (FoM) can be expressed as:

(11) $$FoM=\frac{S_{RI}(1-R_{min})}{FWHM}$$

Here, $R_{min}$ represents the lowest normalized reflection value of the SPR curve.

The limit of detection (LoD) can be calculated as:

(12) $$LoD=\frac{∆n}{∆\theta }\times 0.005{}^{\circ}$$

The Comprehensive Sensitivity Factor (CSF) ratio can be calculated:

(13) $$CSF=\frac{S_{RI}\times (R_{max}-R_{min})}{FWHM}$$

$R_{max}$ represents the maximum reflectance before resonance, typically at non-resonant wavelengths or angles. To ensure high accuracy and numerical reliability, our simulations were performed using a data sampling of 1.0 × 10^5^ points for each reflectance curve, allowing for a smooth and well-defined response.

### 2.3. Biosensor Architecture

**Figure 1:**
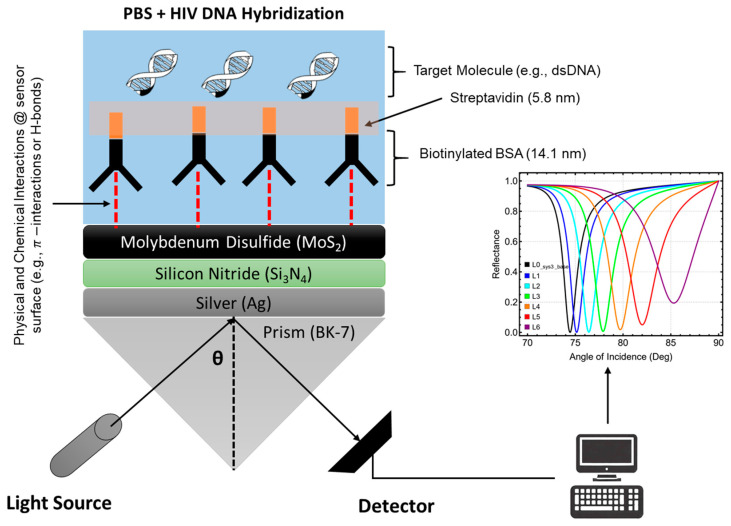
Schematic representation of the proposed SPR-based biosensor for hybridized HIV DNA binding on the MoS_2_/Si_3_N_4_ layer. L0 represents the baseline systems in PBS, and L1–L6 (one to six layers) refers to the number of MoS_2_ layers involved in the sensing process, considering the refractive index of the complex BSA + Strep. + dsDNA.

Table 1 summarizes the SPR sensor configurations analyzed in this study, each incorporating different material layers (Figure 1). The reference system, Sys_0_ (P/Ag/M_PBS_), consists of a prism and a silver (Ag) thin film immersed in a phosphate-buffered saline (PBS) medium, serving as the baseline structure. Sys_1_ (P/Ag/M_PBS+HIV_) replaces the PBS medium with an HIV DNA-containing PBS solution, enabling the study of resonance shifts induced by DNA hybridization. Sys_2_ (P/Ag/SN/M_PBS+HIV_) introduces a silicon nitride (Si_3_N_4_) dielectric layer. Sys_3_ (P/Ag/SN/MoS_2_/M_PBS + HIV) further incorporates a molybdenum disulfide (MoS_2_) monolayer, which is expected to optimize resonance shift, sensitivity, and spectral resolution.

Our SPR sensor differs from the dual-channel design [8] by operating under a thermally controlled environment, eliminating the need for Polydimethylsiloxane (PDMS) for temperature compensation. PDMS was used to track and correct temperature-induced refractive index variations, but in our approach, stable conditions are assumed to ensure that resonance angle shifts result solely from HIV DNA hybridization, making an independent temperature-sensitive layer unnecessary.

Additionally, we adopt the same approach as in [8], where the refractive index (RI = 1.340) corresponds to a modified PBS solution containing biomolecules that are essential for HIV DNA hybridization. This approach ensures consistency in modeling the resonance angle shifts, allowing us to evaluate sensor performance under well-defined conditions. The baseline refractive index (1.335) represents pure PBS (Table 2), a widely used biosensing medium due to its stable pH, ionic strength, and ability to maintain biomolecular interactions [33]. PBS minimizes non-specific adsorption and preserves DNA structure, making it an ideal control medium. The transition from 1.335 to 1.340 occurs due to the addition of biomolecular components, including biotinylated bovine serum albumin (b-BSA), streptavidin, biotinylated probe DNA, and HIV target DNA, all of which contribute to the refractive index shift upon hybridization. The other initial parameters (i.e., refractive indices and thicknesses) used in the current study are reported in Table 2.

## 3. Results and Discussions

### 3.1. Selecting the Best Configurations

**Figure 2:**
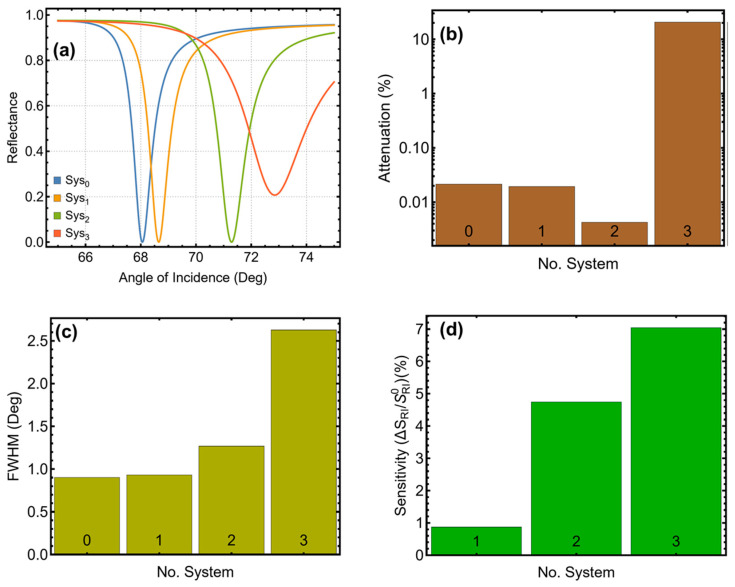
Performance analysis of the proposed SPR sensor configurations (Sys_0_ to Sys_3_). (**a**) Reflectance curves as a function of the angle of incidence for each system. (**b**) Attenuation percentage of the SPR curve and (**c**) FWHM of the SPR dip for each configuration. (**d**) Sensitivity enhancement of Sys_1_–Sys_3_ relative to Sys_0_.

The results presented in Figure 2 and Table S1 provide insight into the impact of Si_3_N_4_ and MoS_2_ on the SPR sensor’s performance for HIV DNA hybridization detection. The SPR peak position (Figure 2a), representing the resonance angle, shows a progressive shift with the addition of layers. Sys_0_, the baseline system, exhibits a resonance angle of 68.06°, while the introduction of HIV DNA in Sys_1_ leads to a slight shift to 68.65°. A more pronounced shift occurs in Sys_2_ (71.29°) with the inclusion of Si_3_N_4_, and the largest shift is observed in Sys_3_ (72.85°) due to MoS_2_ integration. This trend confirms that Si_3_N_4_ and MoS_2_ effectively alter the local refractive index, thereby enhancing plasmonic interactions and improving biosensing performance.

The attenuation percentage (Figure 2b), indicative of plasmonic losses, varies across configurations. Sys_0_ and Sys_1_ show minimal losses at 0.02% and 0.02%, respectively, while a significant reduction is observed in Sys_2_ (0.004%), highlighting the role of Si_3_N_4_ in minimizing optical losses. In contrast, Sys_3_ exhibits a notably higher attenuation of 20.705%, which can be attributed to MoS_2_-induced absorption, affecting the overall efficiency of the sensor. Although MoS_2_ enhances resonance effects, its increased plasmonic losses must be carefully considered when optimizing SPR performance.

**Figure 3:**
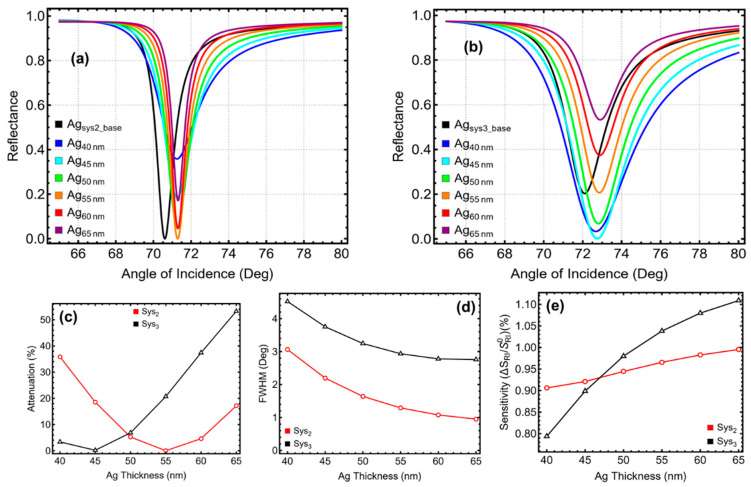
Optimization of the silver (Ag) thickness for Sys_2_ and Sys_3_, evaluating its impact on SPR sensor performance. (**a**) Reflectance curves for Sys_2_ with varying Ag thicknesses, compared to the baseline system (Ag_sys2_base_). (**b**) Reflectance curves for Sys_3_ under the same conditions, referenced against Ag_sys3_base_. (**c**) Attenuation percentage, (**d**) FWHM, and (**e**) sensitivity enhancement of Sys_2_ and Sys_3_ relative to their respective baseline configurations.

Spectral broadening (Figure 3c), measured through FWHM, provides information about resonance dip sharpness. Sys_0_ has the narrowest FWHM at 0.90°, and a slight increase is seen in Sys_1_ (0.93°) due to HIV DNA hybridization effects. The introduction of Si_3_N_4_ in Sys_2_ results in a broader FWHM of 1.27°, which remains within an acceptable range for spectral resolution. However, Sys_3_ exhibits broadening with an FWHM of 2.63°, suggesting that MoS_2_, while improving sensitivity, contributes to a loss in resonance sharpness. This balance between sensitivity and spectral resolution is critical in sensor optimization.

Sensitivity enhancement is measured relative to Sys_0_ (Figure 3d). Sys_1_ shows a marginal improvement of 0.87%, reflecting the impact of HIV DNA presence alone. A substantial increase is seen in Sys_2_ (4.75%), reinforcing the effectiveness of Si_3_N_4_ in boosting plasmonic response. The highest enhancement is observed in Sys_3_ (7.04%), demonstrating the additional advantage provided by MoS_2_. Thus, the selection of Sys_2_ and Sys_3_ for further comparison is justified by their superior resonance shift and sensitivity improvement. In particular, Sys_2_ achieves a balanced trade-off with reduced attenuation and moderate spectral broadening, making it suitable for high-precision biosensing. As well, Sys_3_ exhibits the highest sensitivity but with increased optical losses, which could affect long-term stability.

### 3.2. Optimization: Metal Thin Film

The optimization of silver (Ag) thickness for Sys_2_ and Sys_3_ reveals the importance of balancing plasmonic losses, spectral resolution, and sensitivity enhancement to achieve the best sensing performance (Figure 3 and Table S2). The SPR peak position shows only slight variations across different Ag thicknesses, confirming that adjusting the silver layer primarily fine-tunes the plasmonic response rather than drastically altering resonance conditions (Figure 3a,b). However, the impact on attenuation and spectral broadening is far more pronounced (Figure 3c). In Sys_2_, attenuation decreases sharply with increasing thickness, reaching its lowest value at 55 nm (0.004%), before increasing again at 60 nm (4.60%) and 65 nm (17.14%). This confirms that 55 nm minimizes plasmonic losses, ensuring a well-defined resonance dip with minimal energy dissipation. In Sys_3_, attenuation follows a different trend due to the presence of MoS_2_, remaining low at 45 nm (0.18%) but increasing significantly at 55 nm (20.71%), 60 nm (37.41%), and 65 nm (53.28%). The steep rise in losses beyond 45 nm suggests that thicker silver layers, combined with MoS_2_, lead to excessive energy absorption, reducing sensor efficiency.

Spectral resolution also follows a predictable trend (Figure 3d), with FWHM narrowing as Ag thickness increases, improving resonance sharpness. However, Sys_3_ consistently exhibits broader resonance dips than Sys_2_, reinforcing that MoS_2_ contributes to spectral broadening, which must be carefully controlled. The sensitivity results further justify the choice of 55 nm for Sys_2_ and 45 nm for Sys_3_. While sensitivity increases with thickness (Figure 3e), the improvement beyond 55 nm in Sys_2_ (0.97%) and 45 nm in Sys_3_ (0.90%) is marginal, whereas plasmonic losses rise significantly. This trade-off confirms that these thicknesses provide the best balance between high sensitivity, minimal attenuation, and optimal spectral resolution.

Hence, 55 nm for Sys_2_ ensures minimal plasmonic losses, well-defined resonance characteristics, and strong sensitivity, making it the most stable configuration. A thickness of 45 nm for Sys_3_, on the other hand, prevents excessive attenuation while maintaining a high sensing response, ensuring optimal performance despite the strong light–matter interactions introduced by MoS_2_.

### 3.3. Optimization: Si_3_N_4_

**Figure 4:**
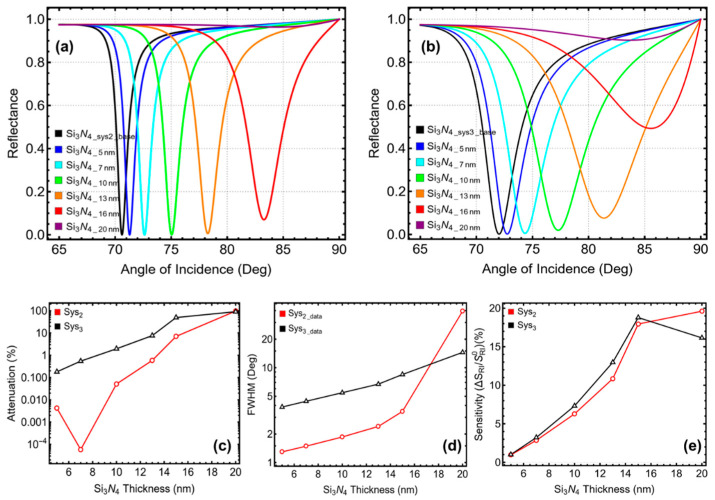
Optimization of the silicon nitride (Si_3_N_4_) thickness for Sys_2_ and Sys_3_, evaluating its impact on SPR sensor performance. (**a**) Reflectance curves for Sys_2_ with varying Si_3_N_4_ thicknesses, compared to the baseline system (Si_3_N_4_sys2_base_). (**b**) Reflectance curves for Sys_3_ under the same conditions, referenced against Si_3_N_4_sys3_base_. (**c**) Attenuation percentage, (**d**) FWHM, and (**e**) sensitivity enhancement of Sys_2_ and Sys_3_ relative to their respective baseline configurations.

The optimization of silicon nitride (Si_3_N_4_) thickness for Sys_2_ and Sys_3_ highlights the importance of selecting a suitable thickness to balance plasmonic performance, energy dissipation, and sensing sensitivity (Figure 4 and Table S3). The SPR peak position shifts significantly with increasing Si_3_N_4_ thickness, confirming its strong influence on resonance conditions (Figure 4a,b). For Sys_2_, the peak moves from 71.29° at 5 nm to 84.45° at 20 nm, while for Sys_3_, it increases from 72.75° at 5 nm to 83.65° at 20 nm. This shift indicates that thicker Si_3_N_4_ layers lead to stronger plasmonic interactions.

Attenuation results confirm that excessive Si_3_N_4_ thickness leads to a rise in plasmonic losses (Figure 4c). In Sys_2_, attenuation remains minimal up to 7 nm (about 0.0%), increases moderately at 13 nm (0.59%), and rises significantly at 16 nm (7.01%) before becoming excessive at 20 nm (96.36%). A similar trend is observed in Sys_3_, where attenuation is 0.18% at 5 nm, remains manageable at 7 nm (0.54%), but increases rapidly beyond 10 nm (1.95%), reaching 49.28% at 16 nm and an overwhelming 90.24% at 20 nm. These results confirm that thinner Si_3_N_4_ layers are necessary to prevent energy dissipation.

Spectral broadening follows a consistent pattern (Figure 4d), with FWHM increasing as Si_3_N_4_ thickness grows. In Sys_2_, it starts at 1.30° at 5 nm, increases gradually to 2.40° at 13 nm, and reaches an extreme 39.50° at 20 nm, making detection unreliable at high thicknesses. Similarly, Sys_3_ shows a progressive FWHM increase, from 3.85° at 5 nm to 6.72° at 13 nm, reaching 14.52° at 20 nm. As noted, the broadening effect is particularly pronounced in Sys_3_.

Sensitivity enhancement is reported in Figure 4e. In Sys_2_, sensitivity steadily increases with thickness, peaking at 13 nm (10.85%), after which it continues rising but at a much lower rate relative to the increase in attenuation. For Sys_3_, sensitivity increases consistently, but the optimal trade-off is observed at 7 nm (3.22%), where performance is maximized without introducing excessive spectral broadening or attenuation. Hence, all these findings show that 13 nm of Si_3_N_4_ in Sys_2_ provides the best combination of high sensitivity, moderate spectral broadening, and controlled attenuation, making it the most effective thickness for this configuration. In contrast, Sys_3_ benefits from a thinner 7 nm layer, maintaining low losses, a well-defined resonance peak, and strong sensitivity enhancement without excessive spectral distortion.

### 3.4. Optimization: MoS_2_ Layers

**Figure 5:**
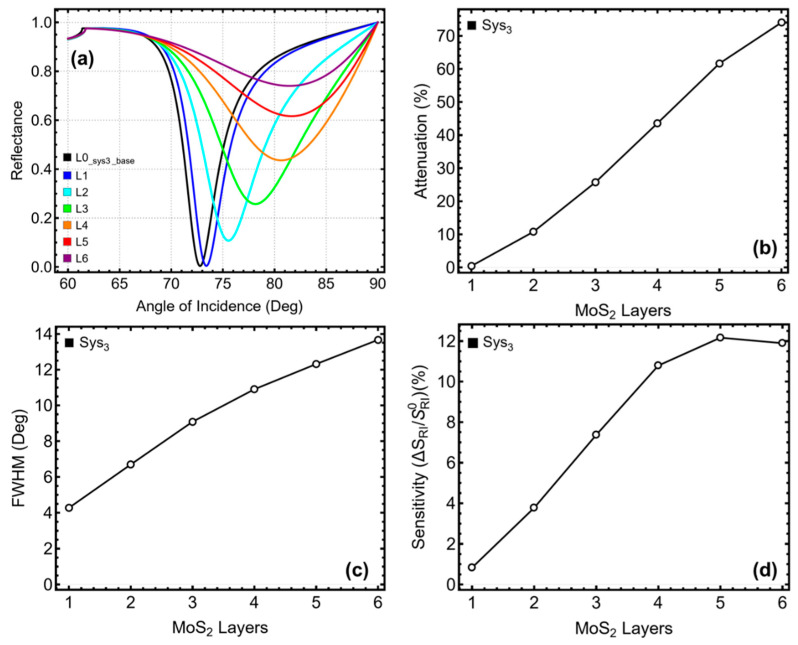
Optimization of the number of molybdenum disulfide (MOS_2_) layers in Sys_3_, evaluating its effect on SPR sensor performance. (**a**) Reflectance curves for Sys_3_ with varying MOS_2_ layers, compared to the baseline system (L0__sys3_base_). (**b**) Attenuation percentage, (**c**) FWHM, and (**d**) sensitivity enhancement relative to the baseline configuration.

The optimization of molybdenum disulfide (MoS_2_) layers in Sys_3_ is shown in Table S4 and Figure 5. The SPR peak position shifts progressively from 73.40° with one layer (L1) to 81.45° with six layers (L6), reflecting the increased refractive index (Figure 5a). However, the shift slows beyond three layers (L3), indicating diminishing benefits. Attenuation remains low at 0.44% with one layer but rises drastically to 10.78% with two layers, 25.74% with three layers, and reaches an impractical 74.03% with six layers, demonstrating the severe plasmonic losses caused by excessive MoS_2_ thickness (Figure 5b).

Spectral broadening follows the same trend (Figure 5c), with FWHM increasing from 4.28° (L1) to 13.67° (L6), reducing the sharpness of the resonance dip and impairing detection accuracy. Sensitivity improves initially (Figure 5d), reaching 12.17% with five layers (L5), but this gain is outweighed by the excessive attenuation and loss of spectral resolution. Beyond four layers (L4), additional MoS_2_ does not significantly enhance sensitivity, confirming that thicker films introduce more drawbacks than benefits. These findings support the selection of monolayer MoS_2_ as the optimal configuration for Sys_3_, ensuring high sensitivity while maintaining low plasmonic losses and sharp resonance characteristics, making it ideal for HIV DNA hybridization detection.

### 3.5. Sensing HIV DNA Hybridization

Table S5 presents the final optimized structural parameters for Sys_2_ and Sys_3_, including refractive index (RI) and thickness values for each material layer. For Sys_2_, the configuration consists of a BK7 prism (RI = 1.5151), a 55 nm silver layer, and a 13 nm silicon nitride layer. In Sys_3_, the silver layer is thinner (45 nm), the Si_3_N_4_ layer is 7 nm, and a monolayer of molybdenum disulfide (MoS_2_, 0.65 nm) is added. These parameters define the final sensor designs for assessing HIV DNA hybridization detection, determining their effectiveness as SPR biosensors.

**Figure 6:**
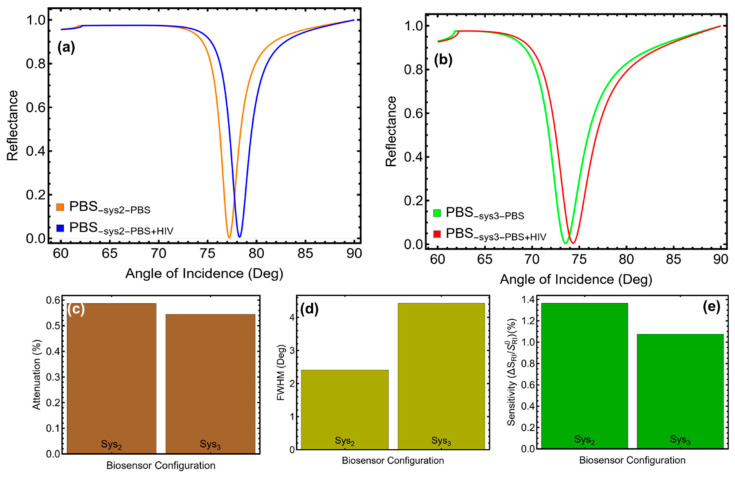
SPR sensor response of Sys_2_ and Sys_3_ for HIV DNA hybridization detection, using the optimized parameters for each configuration. (**a**) Reflectance curves for Sys_2_ comparing the baseline system (PBS__sys2-PBS_) and the system after HIV DNA hybridization (PBS__sys2-PBS+HIV_), illustrating the resonance shift. (**b**) Reflectance curves for Sys_3_ under the same conditions. (**c**) Attenuation percentage for Sys_2_ and Sys_3_, (**d**) FWHM, and (**e**) sensitivity enhancement, highlighting the improved biosensing performance for HIV DNA hybridization detection.

The results in Figure 6 and Table S6 provide a direct comparison of the SPR response of the optimized Sys_2_ and Sys_3_ configurations before (PBS only) and after (PBS + HIV DNA hybridization). The observed resonance shifts confirm the effectiveness of both sensor designs, with Sys_2_ demonstrating a larger shift (Figure 6a), while Sys_3_ exhibits increased spectral broadening due to MoS_2_ integration (Figure 6b). For Sys_2_, the SPR peak shifts from 77.21° to 78.27° after hybridization, indicating a resonance shift of 1.06°. The attenuation increases moderately from 0.30% to 0.59% (Figure 6c), while FWHM remains relatively stable between 2.28° and 2.41° (Figure 6d), ensuring a well-defined resonance dip. The sensitivity enhancement reaches 1.37% (Figure 6e), highlighting the role of Si_3_N_4_ in improving plasmonic confinement without excessive optical losses.

To further remark, in Sys_3_, the resonance shift is slightly lower, moving from 73.55° to 74.34° (0.79° shift), reflecting a reduced sensitivity compared to Sys_2_. The attenuation increases from 0.45% to 0.55%, and FWHM broadens from 4.30° to 4.43°, confirming that while MoS_2_ enhances sensitivity, it also introduces higher plasmonic losses and wider resonance dips. Sensitivity enhancement reaches 1.08%, which is slightly lower than that of Sys_2_.

These findings evidence the unique contributions of Si_3_N_4_ and MoS_2_ in improving SPR-based biosensing. Sys_2_ exhibits superior spectral sharpness and a higher resonance shift, making it the most effective configuration for detecting HIV DNA hybridization. Meanwhile, Sys_3_ benefits from MoS_2_-enhanced sensitivity but at the cost of increased spectral broadening and higher losses, reinforcing that material selection must balance sensitivity and optical performance to optimize biosensor efficiency.

### 3.6. Performance Metrics of SPR Biosensor

**Figure 7:**
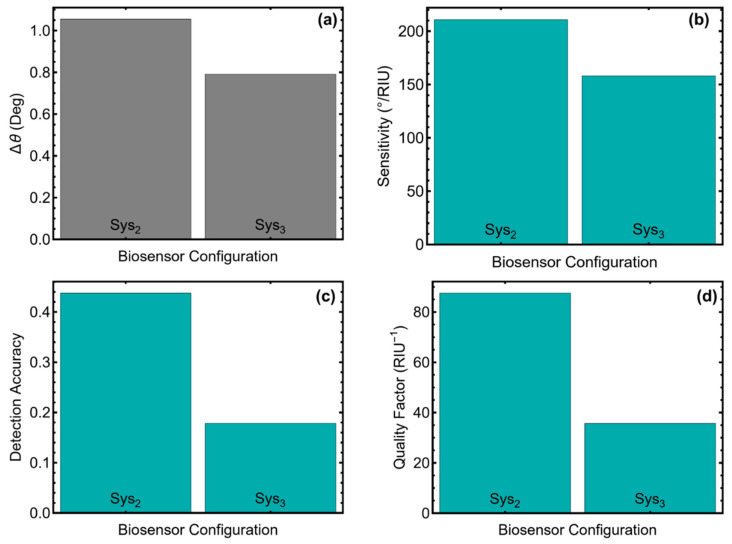
Performance evaluation of optimized Sys_2_ and Sys_3_ after HIV DNA hybridization detection, focusing on key biosensing metrics. (**a**) Resonance angle shift (Δθ), (**b**) sensitivity to refractive index changes, (**c**) detection accuracy, and the (**d**) quality factor.

The results in Figure 7 and Table 3 provide a comprehensive assessment of key biosensing performance metrics for optimized Sys_2_ and Sys_3_ after detecting HIV DNA hybridization. The comparison is based on four essential parameters: resonance angle shift (Δθ) (Figure 7a), sensitivity (S) (Figure 7b), detection accuracy (DA) (Figure 7c), and quality factor (QF) (Figure 7d). Then, Sys_2_ outperforms Sys_3_ on all critical metrics, confirming its superior performance for HIV DNA hybridization detection. The resonance angle shift for Sys_2_ is 1.05°, which is significantly larger than the 0.79° shift in Sys_3_, indicating a more pronounced response to biomolecular binding events. This larger shift translates directly into higher sensitivity, where Sys_2_ achieves 210.9°/RIU, compared to 158.1°/RIU in Sys_3_, demonstrating its enhanced ability to detect small refractive index changes.

Detection accuracy follows the same trend, with Sys_2_ exhibiting a DA of 0.44, which is more than twice that of Sys_3_ (0.18), reinforcing its higher precision in identifying HIV DNA hybridization events. Additionally, the quality factor (QF) of Sys_2_ reaches 87.49 RIU^−1^, which is more than double that of Sys_3_ (35.67 RIU^−1^), indicating a sharper and better-defined resonance dip, which is crucial for minimizing background noise and improving signal clarity.

Despite Sys_3_ incorporating MoS_2_ for additional plasmonic enhancement, the results suggest that the increased optical losses and spectral broadening introduced by MoS_2_ compromise overall sensor performance. While Sys_3_ remains a viable option, Sys_2_ demonstrates superior sensitivity, precision, and resonance stability, making it the most effective configuration for HIV DNA hybridization detection.

**Figure 8:**
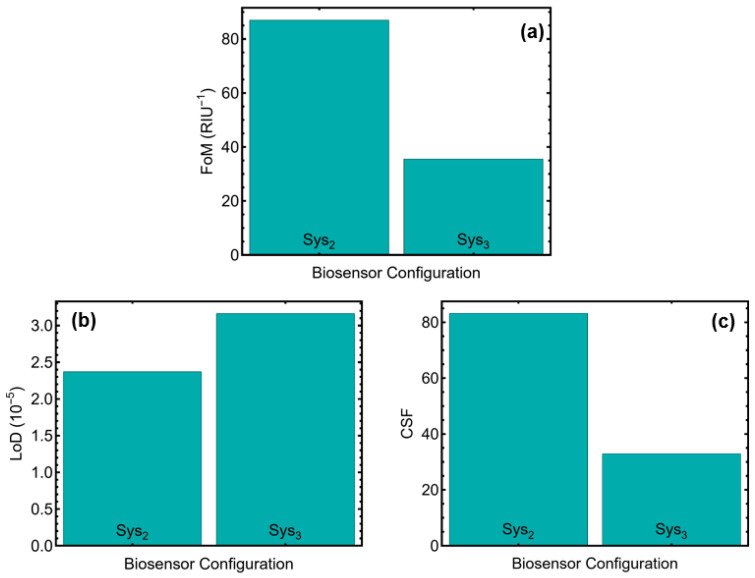
Evaluation of the optimized Sys_2_ and Sys_3_ configurations for HIV DNA hybridization detection, focusing on additional performance metrics. (**a**) Figure of merit (FOM), (**b**) limit of detection (LOD) in 10^−5^, and (**c**) Comprehensive Sensitivity Factor (CSF).

The results in Figure 8 and Table 4 further assess the biosensing performance of Sys_2_ and Sys_3_, focusing on additional key metrics: the figure of merit (FoM) (Figure 8a), the limit of detection (LoD) (Figure 8b), and the Comprehensive Sensitivity Factor (CSF) (Figure 8c). These indicators provide a more detailed understanding of the trade-offs between sensitivity, stability, and selectivity in both configurations. Particularly, Sys_2_ demonstrates superior performance across all evaluated metrics, reinforcing its role as the optimal configuration for HIV DNA hybridization detection. The figure of merit (FoM), which quantifies the balance between sensitivity and spectral resolution, is significantly higher for Sys_2_ (86.98 RIU^−1^) compared to Sys_3_ (35.48 RIU^−1^). This suggests that Sys_2_ maintains a sharper resonance dip while providing strong sensitivity, which is a crucial factor for minimizing detection errors.

The limit of detection (LoD), representing the lowest analyte concentration that can be reliably detected, is slightly better in Sys_3_ (3.16 × 10^−5^) compared to Sys_2_ (2.37 × 10^−5^). This indicates that Sys_3_ benefits from MoS_2_-enhanced sensitivity, enabling detection at slightly lower concentrations. However, this advantage comes at the cost of plasmonic losses and spectral broadening, as previously discussed.

The Comprehensive Sensitivity Factor (CSF), which evaluates overall sensor efficiency by considering sensitivity, stability, and selectivity, further supports Sys_2_ as the superior configuration, with a value of 83.12, which is more than double that of Sys_3_ (32.86). This highlights that despite MoS_2_’s contribution to sensitivity, Sys_2_ provides a more stable and selective sensing response, making it the most balanced and efficient biosensor design.

**Figure 9:**
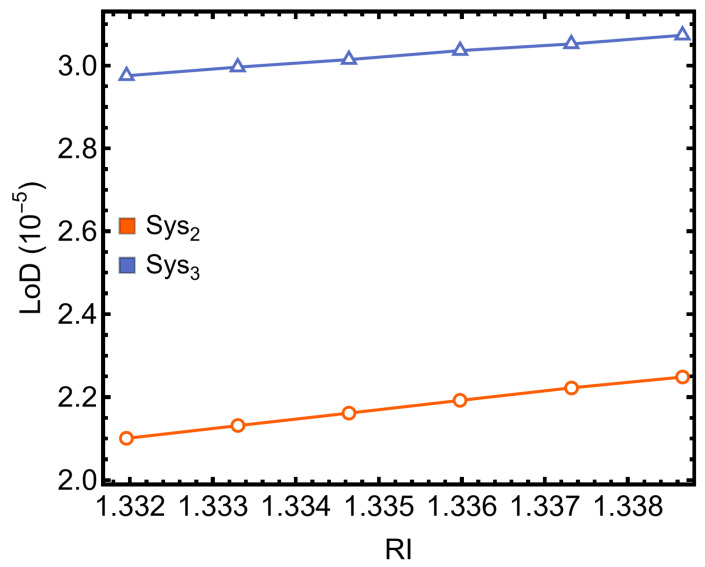
Limit of detection (LoD) as a function of different refractive indexes (RIs), considering up to a variation of ±0.6% in the refractive index by changing the concentration.

Figure 9 presents the limit of detection (LoD) as a function of the refractive index (RI), providing a standard curve to validate the LoD calculation. Since the refractive index of the sensing medium is directly influenced by the concentration, in this case, of HIV DNA hybridization, we followed the methodology reported in Ref. [36], where RI variations of up to ±0.4% were linked to changes in the analyte concentration of DNA hybridization. To extend the analysis, we considered a variation of up to ±0.6% to show a more comprehensive evaluation of LoD trends. The results demonstrate a linear relationship between the LoD (Equation (12)) and the refractive index, confirming the robustness of our detection model. Sys_2_ consistently achieves a lower LoD compared to Sys_3_, reinforcing its higher sensitivity and precision in detecting small analyte concentrations. The linear trend observed in both configurations further validates the reproducibility of our LoD estimations, supporting the feasibility of our numerical approach.

The comparison in Table 5 highlights how the optimized Sys_2_ and Sys_3_ perform against some of the most advanced SPR sensors reported in the literature. The exceptional sensitivity of Sys_2_ (210.9°/RIU) not only surpasses that of all previously reported designs but also emphasizes the effectiveness of Si_3_N_4_ in enhancing plasmonic performance, making it a highly promising biosensor for HIV DNA hybridization detection. Among existing designs, the Ag-ZnSe-based sensor achieves a sensitivity of 208.0°/RIU, which is slightly lower than that of Sys_2_, confirming that the Ag-Si_3_N_4_ combination provides better plasmonic field confinement and stronger light–matter interactions. Compared to Au-MoS_2_-graphene-based sensors, which reach sensitivities of 89.29°/RIU and 130.0°/RIU, Sys_2_ and Sys_3_ show a clear advantage, demonstrating that the integration of Si_3_N_4_ and MoS_2_ offers a more effective strategy for boosting SPR sensor response.

The Au-WSe_2_-graphene-based sensor, with a sensitivity of 178.87°/RIU, also falls short of Sys_2_, further reinforcing the significance of Si_3_N_4_ in improving detection sensitivity. While Sys_3_ (158.1°/RIU) performs well, it does not surpass all previously reported values, likely due to the additional plasmonic losses introduced by MoS_2_, which, while beneficial for sensitivity, also contribute to broader resonance dips and increased optical losses.

These results confirm that Sys_2_ is one of the most sensitive SPR biosensors reported to date. The competitive performance of Sys_3_ shows that MoS_2_ can be a valuable addition for further sensitivity improvements, though careful optimization is needed to minimize trade-offs in optical losses. Overall, Sys_2_ stands out as a next-generation biosensor, capable of achieving state-of-the-art sensitivity while maintaining spectral clarity and detection precision, making it a powerful tool for HIV DNA hybridization detection and broader biosensing applications.

## 4. Discussions

The numerical outcomes of Sys_2_ and Sys_3_ demonstrate significant enhancements in sensitivity, resonance stability, and detection accuracy. However, translating these computational results into realistic HIV detection scenarios requires careful consideration of practical implementation, sample preparation, and integration into clinical workflows. SPR biosensors have already proven their potential in POC diagnostics, but existing limitations, including cost, fragility, and operational complexity, have hindered their widespread adoption. The proposed Sys_2_ and Sys_3_ configurations introduce Si_3_N_4_ and MoS_2_, materials that not only enhance sensitivity but also contribute to practical usability. Si_3_N_4_ offers excellent chemical stability, compatibility with CMOS technology, and low optical loss, making it a suitable choice for miniaturized, cost-effective biosensing platforms. The integration of MoS_2_ in Sys_3_ further enhances plasmonic interactions, though its trade-offs in spectral broadening must be addressed in experimental setups to avoid excessive signal degradation.

The resonance angle shifts of Sys_2_ (1.05°) and Sys_3_ (0.79°) correspond to highly detectable refractive index changes, suggesting that these sensors could reliably distinguish HIV DNA hybridization events even at low concentrations. Compared to traditional techniques like PCR or ELISA, which require trained personnel, time-consuming protocols, and expensive reagents, SPR-based detection offers a label-free, real-time alternative with the potential for rapid field deployment. The lower limit of detection (LoD) values of Sys_2_ (2.37 × 10^−5^) and Sys_3_ (3.16 × 10^−5^) confirm their feasibility for ultra-low-concentration biomolecular sensing, making them particularly relevant for early-stage HIV detection where conventional methods may struggle with sensitivity constraints.

Moreover, the practical integration of these optimized sensors into lab-on-a-chip devices could enable fully automated HIV screening in remote or resource-limited settings. The high stability of Si_3_N_4_ ensures that Sys_2_ could be implemented in long-term clinical monitoring, while Sys_3_’s MoS_2_-enhanced sensitivity suggests potential applications in detecting minimal viral loads, which is crucial in monitoring antiretroviral therapy effectiveness.

## 5. Conclusions

This work explored the integration of Si_3_N_4_ and MoS_2_ into SPR biosensors to enhance the detection of HIV DNA hybridization. Through systematic optimization, Sys_2_ emerged as the most effective configuration, balancing high sensitivity, minimal optical losses, and sharp resonance characteristics. The introduction of MoS_2_ in Sys_3_ improved sensitivity at the cost of increased attenuation and spectral broadening, limiting its overall efficiency. Comparative analysis with existing SPR sensors confirmed the superior performance of Sys_2_, which surpassed widely studied metal-dielectric configurations. These findings provide a theoretical foundation for future experimental validation and demonstrate the potential of hybrid dielectric-plasmonic structures in advancing biosensing technologies for disease diagnostics.

## Figures and Tables

**Table 1:** Summary of the different SPR sensor configurations considered in this study.

Sys No.	Code	Full Name	Nickname
0	Sys_0_	Prism/Silver/PBS Medium	P/Ag/M_PBS_
1	Sys_1_	Prism/Silver/PBS + HIV Medium	P/Ag/M_PBS+HIV_
2	Sys_2_	Prism/Silver/Si_3_N_4_/PBS + HIV Medium	P/Ag/SN/M_PBS+HIV_
3	Sys_3_	Prism/Silver/Si_3_N_4_/Molybdenum Disulfide/PBS + HIV Medium	P/Ag/SN/MoS_2_/M_PBS+HIV_

**Table 2:** Initial parameters used in the SPR sensor model, including the refractive index and thickness of each material.

Material	Refractive Index	Thickness (nm)	Ref.
BK-7 (P)	1.5151	---	[34]
Silver (Ag)	0.056253 + 4.2760 i	55.0	[35]
Si_3_N_4_ (SiN)	2.0394	5.00	[19]
Molybdenum disulfide (MoS_2_)	5.0805 + 1.1723 i	0.65	[31]
PBS (M)	1.335	---	[8]
PBS + HIV DNA hybridization (BSA + Strep. + dsDNA)	1.340	---	[8]

**Table 3:** Numerical results corresponding to the performance metrics shown in Figure 7 for optimized Sys_2_ and Sys_3_ after HIV DNA hybridization detection.

Configuration	∆θ	S (°/RIU)	DA	QF (*RIU*^−1^)
**Sys_2-PBS+HIV_**	1.054	210.9	0.437	87.493
**Sys_3-PBS+HIV_**	0.791	158.1	0.178	35.674

**Table 4:** Numerical results corresponding to the performance metrics presented in Figure 8 for optimized Sys_2_ and Sys_3_ after HIV DNA hybridization detection.

Configuration	FoM (*RIU*^−1^)	LoD (10^−5^)	CSF
**Sys_2-PBS+HIV_**	86.979	2.370	83.121
**Sys_3-PBS+HIV_**	35.480	3.163	32.862

**Table 5:** Comparison of the maximum sensitivity of the optimized sensor (Sys_2_ and Sys_3_) with those available in the literature.

Configuration	S (°/RIU)	Ref. #
Ag-ZnSe-based sensor	208.0	[8]
Au-MoS_2_-graphene-based sensor	89.29	[37]
Au-MoS_2_-graphene-based sensor	130.0	[38]
Au-WSe_2_-graphene-based sensor	178.87	[39]
Ag-Si_2_N_4_-based sensor (Sys_2_)	210.9	This work
Ag-Si_2_N_4_-MoS_2_-based sensor (Sys_3_)	158.1	This work

## Data Availability

The original contributions presented in the study are included in the article/Supplementary Materials; further inquiries can be directed to the corresponding author.

## Supplementary Materials

The following supporting information can be downloaded at: www.mdpi.com/article/10.3390/mi16030295/s1, Table S1: Numerical results for the SPR peak position, attenuation, FWHM, and sensitivity enhancement for each system (Sys_0_ to Sys_3_); Table S2: Numerical results for the SPR peak position, attenuation, FWHM, and sensitivity enhancement as a function of silver (Ag) thickness for Sys_2_ and Sys_3_; Table S3: Numerical results for the SPR peak position, attenuation, FWHM, and sensitivity enhancement as a function of silicon nitride thickness for Sys_2_ and Sys_3_; Table S4: Numerical results for the SPR peak position, attenuation, FWHM, and sensitivity enhancement as a function of the number of the molybdenum disulfide layers for Sys_2_ and Sys_3_; Table S5: Optimized parameters of Sys_2_ and Sys_3_ configurations, and refractive index (RI) of HIV DNA hybridization in PBS; Table S6: Numerical results for the SPR peak position, attenuation, FWHM, and sensitivity enhancement as a function of the different systems for HIB DNA hybridization.
