# Supplementary material for: The Effect of MoS2 and Si3N4 in Surface Plasmon Resonance Biosensors for HIV DNA Hybridization Detection: A Numerical Study
Source: Micromachines (Basel). 2025 Feb 28;16(3):295. doi: 10.3390/mi16030295 (PMC11946481; doi:10.3390/mi16030295)
Supplement: Supplementary file 1 [file micromachines-16-00295-s001.zip › micromachines-3499210-supplementary.pdf]

Article

The effect of MoS_2 and Si_3N_4 in Surface Plasmon Resonance Biosensors for the HIV DNA Hybridization Detection: A Numerical Study

Talia Tene[1,*], Diana Coello-Fiallos[2], María de Lourdes Palacios Robalino[2], Fabián Londo[2], Cristian Vacacela Gomez[3,*]

[1] Department of Chemistry, Universidad Técnica Particular de Loja, Loja 110160, Ecuador
[2] Facultad de Ciencias, Escuela Superior Politécnica de Chimborazo (ESPOCH), Riobamba, 060155, Ecuador
[3] INFN-Laboratori Nazionali di Frascati, Via E. Fermi 54, 00044 Frascati, Italy

Correspondence: tbtene@utpl.edu.ec (T.T.) & cristianisaac.vacacelagomez@utpl.edu.ec (C.V.G.)

Supplementary Tables

Table S1. Numerical results for the SPR peak position, attenuation, FWHM, and sensitivity enhancement for each system (Sys_0 to Sys_3). The SPR peak position represents the resonance angle for each configuration. Attenuation (%) indicates the plasmonic losses, FWHM reflects the spectral broadening of the resonance dip, and Enhancement (%) quantifies the improvement in sensitivity relative to Sys_0.

Sys No.	Code	SPR Peak position	Attenuation (%)	FWHM	Enhancement (%)
0	Sys_0	68.058	0.021	0.901	0.0
1	Sys_1	68.651	0.019	0.931	0.871
2	Sys_2	71.289	0.004	1.268	4.747
3	Sys_3	72.851	20.705	2.627	7.042

Table S2. Numerical results for the SPR peak position, attenuation, FWHM, and sensitivity enhancement as a function of silver (Ag) thickness for Sys_2 and Sys_3. The SPR peak position shifts with increasing thickness, affecting resonance conditions. Attenuation (%) shows the plasmonic losses, which reach a minimum at an optimal thickness. FWHM indicates the spectral broadening, with narrower values corresponding to improved resolution. Enhancement (%) quantifies the relative sensitivity improvement, demonstrating the optimized performance of Sys_2 and Sys_3 at specific Ag thicknesses.

**Thickness (nm)**	**SPR Peak position**	**Attenuation (%)**	**FWHM**	**Enhancement (%)**
		**Sys_2**		
40	71.247	35.860	3.062	0.906
45	71.258	18.558	2.194	0.921
50	71.274	5.226	1.639	0.943
55	71.289	0.004	1.291	0.965
60	71.301	4.601	1.077	0.982
65	71.310	17.139	0.951	0.995
		**Sys_3**		
40	72.674	3.349	4.520	0.794
45	72.749	0.180	3.752	0.899
50	72.808	6.845	3.244	0.979
55	72.850	20.705	2.934	1.038
60	72.880	37.411	2.778	1.079
65	72.901	53.283	2.757	1.109

Table S3. Numerical results for the SPR peak position, attenuation, FWHM, and sensitivity enhancement as a function of silicon nitride thickness for Sys_2 and Sys_3.

**Thickness (nm)**	**SPR Peak position**	**Attenuation (%)**	**FWHM**	**Enhancement (%)**
		**Sys_2**		
5	71.288	0.004	1.296	0.966
7	72.618	0.000	1.488	2.850
10	75.043	0.051	1.860	6.284
13	78.266	0.586	2.404	10.848
16	83.279	7.008	3.461	17.948
20	84.451	96.356	39.503	19.608
		**Sys_3**		
5	72.750	0.180	3.851	1.008
7	74.341	0.544	4.429	3.217
10	77.296	1.950	5.452	7.321
13	81.366	7.602	6.715	12.971
16	85.553	49.278	8.482	18.783
20	83.653	90.244	14.521	16.145

Table S4. Numerical results for the SPR peak position, attenuation, FWHM, and sensitivity enhancement as a function of the number of the molybdenum disulfide layers for Sys_2 and Sys_3.

**Layers**	**SPR Peak position**	**Attenuation (%)**	**FWHM**	**Enhancement (%)**
L1	73.396	0.439	4.277	0.830
L2	75.540	10.778	6.696	3.775
L3	78.153	25.744	9.082	7.364
L4	80.649	43.588	10.902	10.793
L5	81.651	61.659	12.314	12.170
L6	81.453	74.032	13.669	11.898

Table S5. Optimized parameters of Sys_2 and Sys_3 configurations, and refractive index (RI) of HIV DNA hybridization in PBS

Material	Refractive Index (RI)	Thickness (nm)
	**Sys_2**	
BK7 (P)	1.5151	---
Ag	0.056253 + 4.2760 i	55.0
Si_3N_4 (SiN)	2.0394	13.0
	**Sys_3**	
BK7 (P)	1.5151	---
Ag	0.056253 + 4.2760 i	45.0
Si_3N_4 (SiN)	2.0394	7.0
Molybdenum Disulfide (MoS_2)	5.0805 + 1.1723i	0.65*L (L=1)
	**HIV in PBS solution**	
HIV genome DNA (BSA + Strep. + dsDNA)@25 ºC	1.340	---

Table S6. Numerical results for the SPR peak position, attenuation, FWHM, and sensitivity enhancement as a function of the different systems for HIB DNA hybridization.

Configuration	SPR Peak position	Attenuation %	FWHM	Enhancement (%)
**Sys_{2-PBS}**	77.212	0.302	2.284	0.0
**$Sys_{2-PBS+HIV}$**	78.266	0.586	2.410	1.365
**Sys_{3-PBS}**	73.550	0.454	4.303	0.0
**$Sys_{3-PBS+HIV}$**	74.341	0.545	4.432	1.075
